# SSRIs-induced liver injury adverse events: a data-based retrospective study and investigation of potential toxicological mechanisms

**DOI:** 10.3389/fphar.2026.1842778

**Published:** 2026-07-06

**Authors:** Jianru Wu, Chufeng Ding, Yicong Ma, Qiaoying Huang, Qimin Wu, Zhenpo Zhang, Yankun Liang, Jingping Zheng, Wenyu Wu, Xiaoyu Liu, Fenfang Wei, Qian Wang, Ling Su

**Affiliations:** 1 Shenzhen Institute of Pharmacovigilance and Risk Management, Shenzhen, Guangdong, China; 2 College of Pharmacy, Jinan University, Guangzhou, Guangdong, China

**Keywords:** adverse event signal detection, drug-induced liver injury, FAERS, pharmacovigilance, protein-protein interaction, selective serotonin reuptake inhibitors, signaling pathways, updated RUCAM

## Abstract

**Aim:**

To systematically evaluate safety signals of selective serotonin reuptake inhibitors (SSRIs) associated with drug-induced liver injury (DILI) using the FAERS database, and to explore potential toxicological mechanisms through drug-gene interaction network analysis.

**Methods:**

Adverse event data for six SSRIs (2013–2023) were extracted from FAERS. Descriptive analysis and disproportionality signal detection (ROR, BCPNN) were performed. Time to DILI onset and factors influencing mortality were analyzed. Network pharmacology explored drug-gene interactions. Due to the absence of individual-level clinical data in FAERS, we could not apply the updated RUCAM, the gold standard for DILI causality assessment.

**Results:**

DILI reports were most frequent in patients aged 18–64 years and in females. Sertraline had the highest number of reports; fluvoxamine the fewest. Common signals across SSRIs included hepatocellular injury and necrosis. Fluoxetine showed unique signals for hepatic steatosis, paroxetine for chronic active hepatitis, and sertraline for severe liver failure (e.g., primary biliary cholangitis, hemorrhagic hepatic cyst). Median time to onset was within the first month of treatment. Age 18–44 years (OR = 3.03, 95% CI: 1.12–8.18) and low body weight ≤60 kg (OR = 0.45, 95% CI: 0.18–1.14) were significant predictors of mortality. Network analysis suggested shared mechanisms (e.g., CYP450 dysfunction, apoptosis dysregulation) and drug-specific pathways (e.g., PI3K-Akt for sertraline, IL-17 signaling for fluoxetine/fluvoxamine).

**Conclusion:**

DILI safety signals for SSRIs are most frequent within the first month of treatment, particularly in female patients. Considerable variability in hepatotoxicity profiles among SSRIs supports drug-specific monitoring. These hypothesis-generating findings provide a basis for personalized risk assessment and require confirmation in prospective studies.

## 1 Introduction

Depression is the second leading global health burden. Pharmacotherapy remains the cornerstone of treatment and among the available antidepressant classes, selective serotonin reuptake inhibitors (SSRIs) – including fluoxetine, paroxetine, sertraline, citalopram, escitalopram, and fluvoxamine–have become the most widely used agents, accounting for over 70% of antidepressant prescriptions (Thapar et al., 2022). Although SSRIs effectively alleviate depressive symptoms and have favorable safety profile, long-term use may be associated with drug-induced liver injury (DILI) (Machmutow et al., 2019), a severe adverse drug reaction that can progress to acute liver failure. DILI encompasses a broad clinical spectrum, ranging from asymptomatic, transient elevations of liver enzymes (typically alanine aminotransferase and aspartate aminotransferase) that resolve upon drug discontinuation, to rare but life-threatening conditions such as acute hepatitis, fulminant liver failure, and death requiring liver transplantation (Alempijevic et al., 2017). The diagnosis of DILI remains challenging as there is no definitive biomarker. The updated Roussel Uclaf Causality Assessment Method (RUCAM) published in 2016 is the most widely used diagnostic algorithm for DILI (Danan and Teschke, 2015), providing a quantitative scoring system to grade the likelihood that a specific drug caused the observed liver injury. And in 2017, the Chinese Society of Hepatology (CSH) issued its first evidence-based guidelines for the diagnosis and treatment of DILI, which explicitly recommend the sprospective use of the updated RUCAM for causality assessment in clinical practice and research (Yu et al., 2017). However, the detailed clinical and laboratory information required for RUCAM scoring is generally not available in the FDA Adverse Event Reporting System (FAERS). Moreover, many suspected cases may be due to alternative causes rather than the drug itself. As highlighted by Teschke and Danan in their systematic review, more than one-third of suspected DILI cases in published series may be misattributed to drugs due to the presence of alternative causes such as viral hepatitis, autoimmune hepatitis, or ischemic injury (Teschke and Danan, 2018). The hepatotoxic risk associated with antidepressants remains understudied (Anagha et al., 2021). While the overall incidence of SSRI-induced DILI is relatively low, severe complications such as hepatitis and liver dysfunction may occur in individual cases, and the underlying pathogenic mechanisms remain unclear (Dai et al., 2020).

**FIGURE 1:**
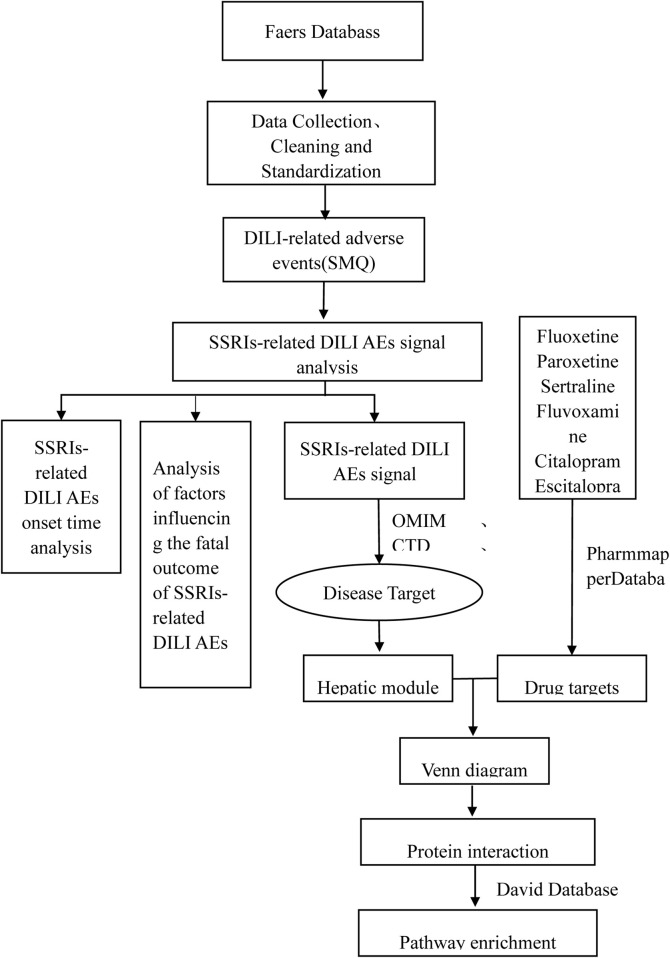
The technical route of this study. Online databases (PharmMapper and Genecards) were used to identify potential targets for AFB1 and liver damage. The intersection targets were confirmed, and the PPI network was constructed. Clustering analysis was used to analyze the network, and GO and KEGG enrichment analysis was performed to predict the functions and pathways involved in AFB1-induced liver damage.

Two critical knowledge gaps persist in current research: our study relies on retrospective FAERS data that lack detailed individual patient information, we are unable to apply RUCAM or similar causality assessments to the individual reports; and inadequate mechanistic integration of clinical observations with molecular pathway analysis. This investigation addresses these limitations through a multi-dimensional analytical framework focusing on six clinically prevalent SSRIs, including escitalopram and sertraline. First, we aimed to systematically evaluate and compare DILI-related adverse event signals for the six most commonly prescribed SSRIs using the FAERS database, focusing on severe liver injury events and analyzing factors such as time to onset and mortality. Second, we sought to explore potential shared and drug-specific toxicological mechanisms using a computational network pharmacology approach that integrates drug-target prediction, protein-protein interaction networks, and pathway enrichment analysis. We also constructed drug-disease protein interaction networks to pinpoint critical biological pathways implicated in hepatotoxicity, as illustrated in Figure 1.

Unlike previous studies relying solely on adverse event reporting, this research bridges real-world pharmacovigilance data with mechanistic exploration. Systematic screening of FAERS risk signals enables early identification of potential hepatotoxicity, while molecular network analysis elucidates both shared and drug-specific pathways underlying SSRI-associated DILI. These findings yield translational implications for clinical practice: informing risk-stratified SSRI selection and optimizing monitoring protocols for patients requiring extended antidepressant therapy. This integrated strategy enhances medication safety through concurrent identification of clinical risk patterns and elucidation of their biological underpinnings.

## 2 Experimental procedures

### 2.1 FAERS data extraction

This study acquired drug adverse event (ADE) reports submitted to the FAERS from the first quarter of 2013 to the fourth quarter of 2023. Each quarterly dataset included drug details (DRUG), patient demographics (DEMO), adverse events (REAC), and outcomes (OUTC) (Shu et al., 2022a). The generic names of the target drugs—citalopram, escitalopram, fluoxetine, fluvoxamine, paroxetine, and sertraline—were predefined, and corresponding data were retrieved from FAERS. All quarterly DEMO, DRUG, REAC, and other report data were cleaned and consolidated (Shu et al., 2022b). Duplicate reports were removed based on CaseID, patient name, age, country, generic/brand names, and primary suspected drug (PS), retaining the most recent entries. This process yielded 110,156 reports related to the six SSRIs. Subsequently, adverse event terms were standardized using MedDRA Preferred Terms (PTs) to refine the selection of SSRI-associated adverse reactions. Severe DILI cases were identified through a narrow search of the Standardized MedDRA Query (SMQ version 27.1; SMQ code: 20000007), encompassing 47 PTs. Duplicate records were excluded from analysis (Masco et al., 2021). The operational definition of DILI in this study included drug-related severe hepatic events, as detailed in Table 1.

**TABLE 1:** Drug-related liver disease - the preferred term for severe event (SMQ) cases only.

NO.	Prefer terms	NO.	Prefer terms
1	Hepatitis fulminant	2	Liver injury
3	Hepatocellular injury	4	Haemangioma of liver
5	Hepatitis acute	6	Liver transplant
7	Hepatic lesion	8	Hepatic cancer
9	Hepatorenal syndrome	10	Hepatic cirrhosis
11	Drug-induced liver injury	12	Non-alcoholic steatohepatitis
13	Hepatitis	14	Hepatic fibrosis
15	Hepatitis toxic	16	Autoimmune hepatitis
17	Hepatotoxicity	18	Haemorrhagic hepatic cyst
19	Mixed liver injury	20	Hepatorenal failure
21	Acute hepatic failure	22	Steatohepatitis
23	Coma hepatic	24	Hepatic cyst
25	Varices oesophageal	26	Hepatic encephalopathy
27	Liver disorder	28	Hepatic neoplasm
29	Hepatic steatosis	30	Cholestatic liver injury
31	Hepatic failure	32	Subacute hepatic failure
33	Hepatitis cholestatic	34	Chronic hepatitis
35	Oesophageal varices haemorrhage	36	Nodular regenerative hyperplasia
37	Benign hepatic neoplasm	38	Biliary cirrhosis
39	Asterixis	40	Liver operation
41	Hepatic necrosis	42	Hepatitis chronic active
43	Hepatic cancer metastatic	44	Focal nodular hyperplasia
45	Ascites	46	Hepatocellular carcinoma
47	Ischaemic hepatitis	​	​

### 2.2 Adverse event signal detection methodology

This study utilized a combined approach integrating the frequency-based Reporting Odds Ratio (ROR) method and the Bayesian Confidence Propagation Neural Network (BCPNN) method to detect adverse event (AE) signals associated with selective serotonin reuptake inhibitors (SSRIs). As described in Table 2, the parameter *a* denotes the number of reports containing both the target drug and the target adverse drug reaction (ADR), *b* represents reports involving the target drug with other ADRs, *c* corresponds to reports of the target ADR with other drugs, and *d* indicates reports of other drugs and other ADRs (Tang et al., 2021). The computational formulas for these two signal detection methods are provided in Table 3. A valid signal was defined when both methods yielded positive results, specifically when the lower limit of the ROR 95% confidence interval (CI) exceeded 1, the target drug’s AE reports numbered at least 3, and the Information Component minus two standard deviations (IC-2SD) was greater than 0 (Hu et al., 2020).

**TABLE 2:** Quadruple table of AEs.

Adverse events	Target drug (SSRI)	Other drugs
TargetAEs	A	C
Non-targetAEs	B	D

**TABLE 3:** Two major algorithms used for signal detection. (ROR >1 and lower 95% CI > 1 indicates disproportional reporting; IC-2SD > 0 indicates signal (97.5% confidence)).

Algorithms	Equation	Criteria
ROR	$\text{ROR}=\frac{\left(a/c\right)}{\left(b/d\right)}=\frac{\text{ad}}{\text{bc}}$	a≥3,lower limit of 95% CI > 1
	$95\%\text{CI}=e^{\ln\left(\text{ROR}\right)\pm 1.96\sqrt{\left(\frac{1}{a}+\frac{1}{b}+\frac{1}{c}+\frac{1}{d}\right)}}$	
BCPNN	IC = $\log_{2}\frac{a\left(a+b+c+d\right)}{\left(a+b\right)\left(a+c\right)}$	a≥3,IC025 > 0
	E (IC) = $\log_{2}\frac{\left(a+\gamma 11\right)\left(a+b+c+d+\alpha \right)\left(a+b+c+d+\beta \right)}{\left(a+b+c+d+\gamma \right)\left(a+b+\alpha 1\right)\left(a+c+\beta 1\right)}$	
	V(IC) = $\frac{1}{{\left(\ln2\right)}^{2}}\left\{\left[\frac{\left(a+b+c+d\right)-a+\gamma -\gamma 11}{\left(a+\gamma 11\right)\left(1+a+b+c+d+\gamma \right)}\right]+\left[\frac{\left(a+b+c+d\right)-\left(a+b\right)+\alpha -\alpha 1}{\left(a+b+\alpha 1\right)\left(1+a+b+c+d+\alpha \right)}\right]+\left[\frac{\left(a+b+c+d\right)-\left(a+c\right)+\beta -\beta 1}{\left(a+c+\beta 1\right)\left(1+a+b+c+d+\beta \right)}\right]\right\}$	
	$\gamma =\gamma 11\frac{\left(a+b+c+d+\alpha \right)\left(a+b+c+d+\beta \right)}{\left(a+b+\alpha 1\right)\left(a+c+\beta 1\right)}$	
	95% CI = E (IC)±2 $\sqrt{V\left(\text{IC}\right)}$	
	α1=β1=1;α=β=2;γ11=1	

### 2.3 Network analysis of SSRIs- DILI gene interactions

Network analysis, a cross-disciplinary approach, investigates drug-biological system interactions at the network level by integrating diverse biological data types—including drug-target interactions, protein-protein interactions, gene expression profiles, and disease associations—into comprehensive network models. In this study, biological entities such as SSRIs, drug targets, genes, and liver injury-associated proteins are represented as nodes within the network, while their interactions are depicted as edges. By applying graph theory and network analysis techniques to scrutinize the structural and functional properties of these networks, we aim to predict potential targets and pathways implicated in SSRI-associated DILI, thereby elucidating the molecular mechanisms underlying this adverse effect (She et al., 2024).

### 2.4 Collection and screening of targets

For potential drug targets, the chemical structures of four drugs were identified from the DrugBank database and literature, visualized using the PubChem database. Drug targets were predicted via the online tool PharmMapper with default parameters, retaining targets with a fit score ≥2.8 and z-score ≥0. Fit score ≥2.8 was selected based on the platform’s recommended threshold (top 10% of normalized fit scores), which achieves 85% sensitivity and 78% specificity in cross-validation studies. Z-score ≥0 was applied to retain targets scoring above the random distribution mean (Wang et al., 2017). To enhance data consistency and comparability, severe liver injury adverse event signals in the FAERS database, originally encoded as MedDRA Preferred Terms (PTs), were standardized to Medical Subject Headings (MeSH) terms, facilitating cross-database searches for disease-associated genes in comprehensive repositories such as OMIM, CTD, and GeneCards (Tang et al., 2024).

For disease targets, a multi-source approach was employed to retrieve liver injury-related genes from OMIM, CTD, and GeneCards. Specific search and filtering criteria were applied: genes with inference scores in the top 5% from CTD and relevance scores ≥3 from GeneCards were prioritized (Tang et al., 2024). Detailed screening metrics and parameters are outlined in Table 4. To improve specificity, viral hepatitis-associated genes identified through OMIM were excluded from the liver injury module. Gene IDs and symbols for both drug and disease targets were standardized using the UniProt database. Strongly disease-relevant genes were further refined by applying the CTD database’s top 5% inference score threshold.

**TABLE 4:** Search entries and inclusion criteria for disease genes in each disease database.

Database	Search for terms	Filter criteria for terms	Targets in uniport
A			
OMIM	“Autoimmune, Hepatitis-Virus”	——	84
	Hepatic Failure-Virus”		
	“Hepatotoxicity-Virus”		
	Chemical And Induced Liver Injury Chromic-Virus		
	“Liver Cirrhosis, Biliary-Virus”		
	“Non-Alcoholic Fatty Liver Disease-Virus”		
	“Liver Injury-Virus”		
GeneCard	“Hepatotoxicity”	Relevance score ≥ 5	24
B			
The Comparative Toxicogenomics Database (CTD)	“Chemical And Drug Induced Liver Injury Chromic”	TOP 5% inference score > 29.32	912
	“Hepatitis”	TOP 5% inference score > 89.71	
	“Hepatic Failure”	TOP 5% inference score > 80.68	
	“Hepatic Failure, Acure”	TOP 5% inference score > 87.51	
	“Liver Cirrhosis, Biliary”	TOP 5% inference score > 34.37	
	“Non-Alcoholic Fatty Liver Disease”	TOP 5% inference score > 129.73	
	Autoimmune Hepatitis	TOP 5% inference score > 24.19	
	“Mass Hepatic Necrosis”	TOP 5% inference score > 55.77	
	“Hepatic Encephalopathy”	TOP 5% inference score > 73.57	
	“End Stage Liver Disease”	TOP 5% inference score > 18.05	

After merging and deduplicating the three data sources (OMIM, GeneCard, and CTD), and removing viral hepatitis-associated genes, the final total number of unique targets is 852.

### 2.5 Gene enrichment and pathway analysis

Gene enrichment analysis was conducted using the DAVID database to explore the core mechanisms underlying SSRI-induced drug-induced liver injury (DILI). The shortest distance (*ds*) between nodes was calculated, where *ds* = 0 indicates overlapping targets between the disease module and the drug (Sherman et al., 2022). For the overlapping network, this study selected intersection targets between the six SSRIs and the liver injury module based on the shortest distance (*ds* = 0). Protein-protein interaction (PPI) networks and functional enrichment analyses (Gene Ontology, GO; Kyoto Encyclopedia of Genes and Genomes, KEGG) were performed on these overlapping targets to identify potential core mechanisms in SSRI-induced hepatotoxicity. Enrichment results with Bonferroni-corrected *p*-values < 0.01 were considered significant. Visualization of the enriched networks was executed using Cytoscape 3.9, revealing distinct pathological pathways associated with SSRI-related liver injury (Menche et al., 2015).

## 3 Result

### 3.1 Descriptive analysis of cases of adverse events related to DILI caused by SSRIs

Among severe drug-induced liver injury (DILI) adverse event reports for the six SSRIs, female patients predominated over males in all agents except fluvoxamine which has an equal ratio of male to female patients. Middle-aged patients (45–64 years) constituted the largest demographic group for escitalopram, paroxetine, and citalopram, accounting for 27.8%, 30.1%, and 26.6% of cases, respectively. Sertraline exhibited the highest proportion of young adults (18–44 years) at 30.2%, while fluoxetine showed comparable representation of both young (18–44 years) and middle-aged (45–64 years) groups at 32.0% each. Regarding reporter roles, physician-reported cases predominated across five SSRIs, excluding fluvoxamine. Hospitalization represented the most frequent outcome for escitalopram (47.2%) and fluvoxamine (55.6%), whereas other medically significant events dominated outcomes for fluoxetine (51.3%), paroxetine (48.3%), sertraline (49.0%), and citalopram (45.0%). Geographically, the majority of reports originated from European and North American countries, with France, the United Kingdom, Germany, and the United States contributing the highest proportions. Notably, Denmark accounted for 19.0% of escitalopram-related reports, ranking second only to France in this subset. The specific results are shown in Table 5.

**TABLE 5:** Characteristics of reports on SSRIs-associated DILIs in the FAERS database.

Characteristics	Escitalopram	Fluvoxamine	Fluoxetine	Paroxetine	Sertraline	Citalopram
Number of reports	252	9	306	236	463	429
Gender						
Male	96 (38.1)	4 (44.4)	97 (31.7)	79 (33.5)	146 (31.6)	124 (28.9)
Female	125 (49.6)	4 (44.4)	187 (61.1)	134 (56.9)	252 (54.4)	250 (58.3)
Unknown	31 (12.3)	1 (11.1)	22 (7.2)	23 (9.7)	65 (14.0)	55 (12.8)
Age						
<18	11 (4.4)	3 (33.3)	41 (11.6)	11 (4.7)	42 (9.4)	11 (2.6)
18-44	42 (16.7)	3 (33.3)	98 (27.7)	55 (23.3)	140 (31.5)	86 (20.0)
45-64	70 (27.8)	0 (0)	98 (27.7)	71 (30.1)	91 (20.4)	114 (26.7)
≥65	56 (22.3)	1 (11.1)	70 (19.8)	57 (24.2)	93 (20.9)	99 (23.1)
Unknown	73 (29.0)	2 (22.2)	37 (10.5)	42 (17.8)	80 (17.9)	119 (27.8)
Reporter						
Physician	93 (36.9)	5 (55.6)	106 (34.6)	74 (31.4)	167 (36.1)	152 (35.5)
Pharmacist	34 (13.5)	2 (22.2)	22 (7.2)	31 (13.1)	49 (10.6)	63 (14.7)
Consumer	24 (9.5)	0 (0)	76 (24.8)	92 (39.0)	63 (13.6)	44 (10.3)
Other healthcare professional	81 (32.1)	0 (0)	61 (20)	20 (8.5)	102 (22.0)	126 (29.4)
Other	20 (7.9)	2 (22.2)	41 (13.4)	19 (8.1)	82 (17.7)	44 (10.3)
Outcome						
Hospitalization	119 (47.2)	5 (55.6)	113 (36.9)	99 (41.9)	174 (37.6)	174 (40.5)
Life-threatening	11 (4.4)	0 (0)	18 (5.9)	4 (1.7)	28 (6.1)	14 (3.3)
Death	6 (2.4)	0 (0)	16 (5.2)	16 (6.8)	29 (6.3)	44 (10.3)
Disability	2 (0)	0 (0)	2 (0.7)	3 (1.3)	5 (1.1)	4 (0.9)
Other	114 (45.2)	4 (44.4)	157 (51.2)	114 (48.3)	227 (49.0)	193 (45.0)
Reported year						
2013	23 (9.1)	0 (0)	18 (5.9)	6 (2.5)	23 (4.9)	13 (3.0)
2014	7 (2.8)	0 (0)	21 (6.9)	1 (0.4)	4 (0.9)	5 (1.2)
2015	11 (4.4)	1 (11.1)	15 (4.9)	18 (7.6)	27 (5.8)	20 (4.7)
2016	22 (8.7)	1 (11.1)	17 (5.5)	16 (6.8)	32 (6.9)	42 (9.8)
2017	22 (8.7)	1 (11.1)	17 (5.5)	17 (7.2)	63 (13.6)	55 (12.8)
2018	49 (19.4)	0 (0)	49 (16.0)	38 (16.1)	49 (10.6)	92 (21.4)
2019	41 (16.3)	3 (33.3)	25 (8.2)	29 (12.3)	83 (17.9)	64 (14.9)
2020	19 (7.5)	0 (0)	21 (6.9)	30 (12.7)	61 (13.2)	31 (7.2)
2021	16 (6.3)	0 (0)	38 (12.4)	25 (10.6)	40 (8.6)	25 (5.8)
2022	18 (7.1)	3 (33.3)	40 (13.1)	25 (10.6)	50 (10.8)	33 (7.7)
2023	24 (9.5)	0 (0)	44 (14.4)	32 (13.5)	31 (6.7)	49 (11.4)
Reported country (top 5)						
FR	89 (35.4)	3 (33.3)	102 (33.3)	108 (45.7)	110 (23.8)	115 (26.9)
DK	48 (19.0)	-	-	-	-	-
USA	30 (11.9)	-	27 (8.8)	-	67 (14.5)	57 (13.3)
ES	18 (7.1)	-	-	9 (3.8)	29 (6.3)	-
IN	10 (3.9)	-	-	-	-	-
CN	-	1 (11.1)	-	-	-	-
DE	-	1 (11.1)	-	-	-	44 (10.3)
UK	-	1 (11.1)	89 (29.1)	54 (22.8)	113 (24.4)	-
JP	-	1 (11.1)	-	-	-	-
CA	-	-	22 (7.2)	-	-	-
PT	-	-	17 (5.6)	-	-	-
IT	-	-	-	14 (5.9)	-	33 (7.7)
NL	-	-	-	9 (3.8)	19 (4.1)	37 (8.6)
Other	57 (22.8)	2 (22.2)	49 (16.0)	42 (17.8)	125 (27.0)	143 (33.4)

### 3.2 Frequency distribution of DILI-related adverse events associated with SSRIs

The top 10 severe hepatobiliary adverse events (AEs) associated with each SSRI, ranked by reporting frequency, are summarized in Table 6. For escitalopram, the five most frequently reported severe liver injuries were hepatocellular injury, drug-induced liver injury (DILI), cholestatic hepatitis, hepatitis, and hepatic disorder. Fluoxetine exhibited the highest reporting rates for DILI, hepatocellular injury, hepatic steatosis, liver injury, and hepatotoxicity. Paroxetine-related reports predominantly featured hepatocellular injury, hepatocellular lysis, DILI, hepatic steatosis, and liver injury. Sertraline was primarily associated with DILI, hepatocellular injury, hepatotoxicity, hepatic steatosis, and liver failure. Citalopram showed high frequencies of DILI, hepatocellular injury, liver injury, liver failure, and cholestatic hepatitis. In contrast, fluvoxamine had the highest reporting rate for hepatocellular injury alone. Excluding fluvoxamine, DILI and hepatocellular injury consistently ranked among the top three reported AEs across the remaining five SSRIs, highlighting their shared hepatotoxic risks.

**TABLE 6:** The top ten adverse events of DILI reported in SSRIs reports.

Escitalopram	N	Fluoxetine	N	Paroxetine	N
Hepatocellular injury	51	Drug-induced liver injury	64	Hepatocellular injury	70
Drug-induced liver injury	44	Hepatocellular injury	55	Hepatic cytolysis	39
Hepatitis cholestatic	31	Hepatic steatosis	34	Drug-induced liver injury	29
Hepatitis	21	Liver injury	33	Hepatic steatosis	21
Liver disorder	21	Hepatotoxicity	26	Liver injury	17
Autoimmune hepatitis	20	Hepatic cytolysis	24	Hepatitis	13
Hepatic cytolysis	16	Ascites	19	Liver disorder	11
Hepatic failure	16	Hepatitis	17	Hepatic failure	10
Mixed liver injury	12	Acute hepatic failure	11	Hepatic encephalopathy	8
Liver injury	10	Hepatic cirrhosis	9	Hepatitis fulminant	7
Sertraline	N	Citalopram	N	Fluvoxamine*	N
Drug-induced liver injury	64	Drug-induced liver injury	76	Hepatocellular injury	3
Hepatocellular injury	53	Hepatocellular injury	70	Drug-induced liver injury	1
Hepatotoxicity	51	Liver injury	48	Hepatic cytolysis	1
Hepatic steatosis	40	Hepatic failure	37	Hepatic steatosis	1
Hepatic failure	38	Hepatitis cholestatic	33	Hepatitis	1
Liver transplant	38	Hepatitis	29	Liver Surgery	1
Hepatic cytolysis	37	Hepatic steatosis	28	Hepatic failure	1
Hepatitis cholestatic	37	Mixed liver injury	28	​	​
Primary biliary cholangitis	29	Liver disorder	27	​	​
Hepatitis	27	Acute hepatic failure	22	​	​

* Only 7 distinct DILI, terms were reported for fluvoxamine due to low total report count (N = 9).

### 3.3 Distribution of adverse event signals of DILI caused by SSRIs

Analysis of severe hepatobiliary adverse events (AEs) associated with six SSRIs in the FAERS database revealed distinct signal profiles (Table 7). Escitalopram exhibited the strongest signal for cholestatic hepatitis (ROR [95%CI] = 5.82, IC025 = 2.23), while fluoxetine showed the highest signal strength for hepatocellular injury (ROR [95%CI] = 2.55, IC025 = 1.28). Paroxetine was most strongly associated with chronic active hepatitis (ROR [95%CI] = 12.89, IC025 = 0.52). For sertraline, hemorrhagic liver cyst displayed the strongest signal via the Reporting Odds Ratio method (ROR [95%CI] = 14.20, IC025 = 1.36), whereas primary biliary cholangitis emerged as the top signal using the Bayesian Confidence Propagation Neural Network approach (ROR [95%CI] = 3.79, IC025 = 1.71). Citalopram demonstrated the strongest signal for mixed liver injury (ROR [95%CI] = 4.76, IC025 = 1.97), highlighting variability in hepatotoxic risk profiles across SSRIs.

**TABLE 7:** The distribution of signals for serious liver disease adverse events (SMQ) related to six SSRIs drugs.

Drug	PT term	N	ROR (95%CI)	IC (IC025)
Escitalopram	Hepatocellular injury	51	2.71	1.36
	Drug-induced liver injury	44	1.24	0.28
	Hepatitis cholestatic	31	5.82	2.23
	Autoimmune hepatitis	20	2.30	1.03
	Hepatic cytolysis	16	1.50	0.47
	Mixed liver injury	12	3.36	1.29
Fluvoxamine	Hepatocellular injury	3	1.45	0.18
Fluoxetine	Drug-induced liver injury	64	1.64	0.68
	Hepatocellular injury	55	2.55	1.28
	Hepatic steatosis	34	1.39	0.43
	Liver injury	33	1.17	0.20
	Hepatic cytolysis	24	2.12	0.96
Paroxetine	Hepatocellular injury	70	4.02	0.03
	Hepatic cytolysis	39	4.53	0.05
	Hepatitis fulminant	7	1.73	0.26
	Hepatitis chronic active	3	12.89	0.52
Sertraline	Hepatocellular injury	53	1.22	0.26
	Hepatic cytolysis	37	1.77	0.76
	Hepatitis cholestatic	37	3.09	1.50
	Primary biliary cholangitis	29	3.79	1.71
	Hepatitis acute	18	1.10	0.09
	Hepatitis fulminant	10	1.16	0.09
	Oesophageal varices haemorrhage	7	4.64	1.17
	Haemorrhagic hepatic cyst	6	14.20	1.36
	Mixed liver injury	3	5.92	0.28
	Non-alcoholic steatohepatitis	3	2.93	0.12
Citalopram	Drug-induced liver injury	76	1.14	0.17
	Hepatocellular injury	70	1.92	0.90
	Liver injury	48	1.04	0.04
	Hepatitis cholestatic	33	3.11	1.49
	Mixed liver injury	28	4.76	1.97
	Autoimmune hepatitis	20	1.14	0.14

### 3.4 Time-to-onset analysis of SSRI-Induced liver injury

**FIGURE 2:**
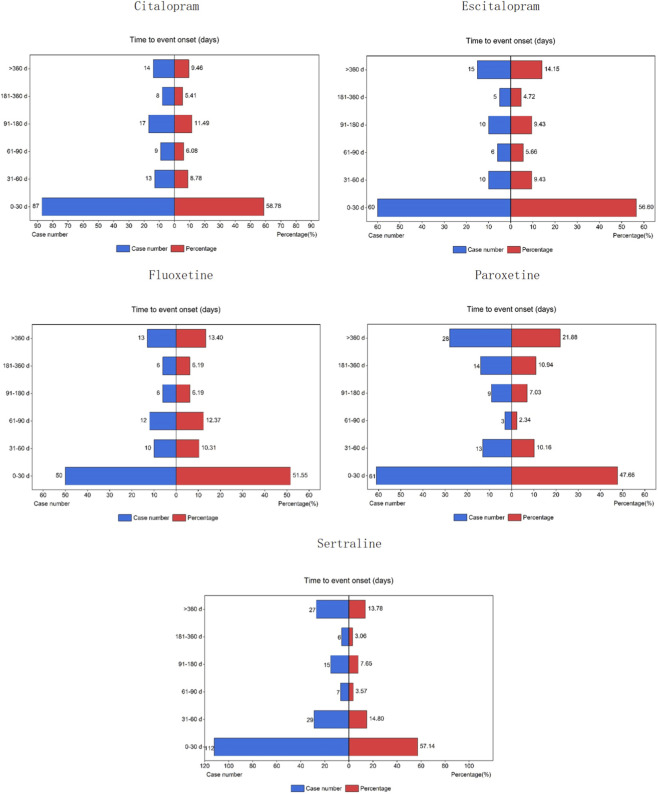
Distribution of time-to-onset for SSRI-associated DILI reports (excluding fluvoxamine, N = 675). The majority of events (63.4%) occurred within the first 30 days of treatment initiation.

This study collected time-to-onset data for adverse events (AEs) associated with five SSRIs from the FAERS database, excluding fluvoxamine due to insufficient sample size for visualization. After removing duplicate and erroneous reports, 106 escitalopram, 97 fluoxetine, 128 paroxetine, 196 sertraline, and 148 citalopram cases provided valid onset time information. As shown in Figure 2, the majority of AEs occurred within the first month of treatment initiation. Delayed onset (beyond 1 year) was observed in 21.88% of paroxetine, 13.4% of fluoxetine, and 14.15% of escitalopram cases. For sertraline, 14.8% of AEs manifested between one to 2 months, while citalopram-associated AEs showed an 11.49% incidence between two to 3 months, reflecting variability in latency periods across SSRIs.

**FIGURE 3:**
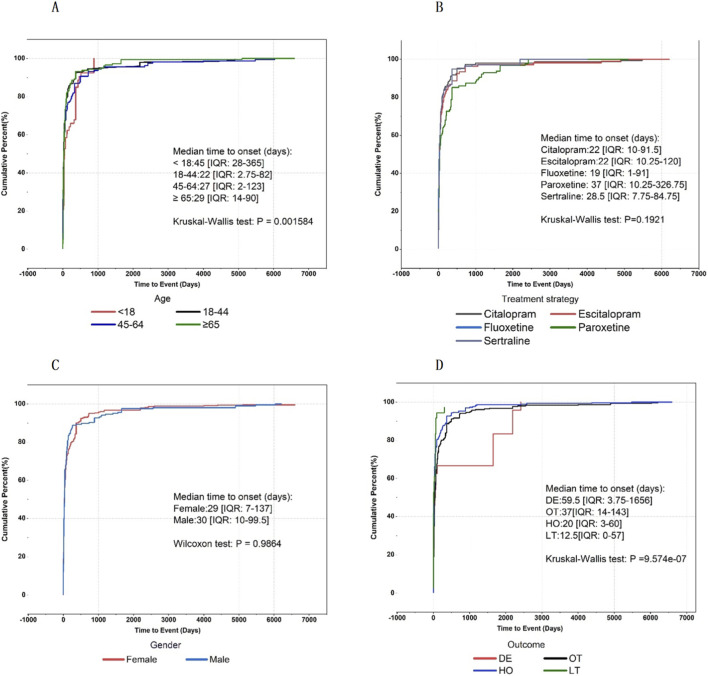
Cumulative distribution curves of different subgroups. (**(A)** Age groups showed significant differences (p < 0.01, Kruskal-Wallis). **(B)** Different SSRIs therapeutic drugs. Individual SSRIs showed variable latency (p = 0.09). **(C)** Gender. Sex showed no significant difference (p = 0.68). **(D)** Outcome. Outcome severity was associated with onset time (p < 0.01)).

As illustrated in Figure 3, the cumulative distribution curves demonstrate the time to onset of SSRIs-associated drug-induced liver injury (DILI) across different subgroups (A. Age B. Sex C. Outcome D. Specific SSRIs agents). Statistical analyses were performed using nonparametric Wilcoxon rank-sum test and Kruskal-Wallis rank-sum test. Significant differences in onset time were observed among age groups (P < 0.01), with the 18-44 age group exhibiting the shortest median onset time (22 days), followed by the 45-64 group (27 days) and the over-65 group (29 days), while the under-18 group showed the longest median onset time (45 days). Similarly, outcome subgroups revealed statistically significant variations (P < 0.01), where the mortality group demonstrated the longest median onset time (59.5 days), succeeded by other serious adverse event outcomes (37 days) and hospitalization cases (20 days), with life-threatening adverse events presenting the shortest median onset time (12.5 days).

Notably, this study failed to detect significant effects of sex or specific SSRI agents on the time to DILI onset. Among the four SSRIs with median onset times below 30 days, citalopram and escitalopram both showed 22-day medians, fluoxetine 19 days, and sertraline 28.5 days. Paroxetine exhibited the longest median onset time at 37 days. Regarding sex-based comparisons, minimal difference was observed between male (30-day median) and female (29-day median) onset times for DILI development.

### 3.5 Univariate logistic regression analysis of fatal outcomes in SSRI-associated DILI

Patients were stratified into mortality and non-mortality groups based on DILI outcomes to evaluate the influence of age, sex, body weight, and dosage on clinical endpoints, as summarized in Table 8. Predictive analysis identified age and body weight as significant determinants of mortality. In age-stratified comparisons, patients aged 18-44 years demonstrated a 3.026-fold higher mortality risk than those aged 45-64 years (OR = 3.026, P < 0.05). Regarding body weight, individuals below 60 kg exhibited a 54.7% reduction in mortality risk compared to those weighing 61-80 kg (OR = 0.453, P < 0.05). Neither sex nor administered dosage showed statistically significant associations with mortality outcomes in drug-induced liver injury cases.

**TABLE 8:** Analysis of factors affecting the mortality of patients with DILI.

Factors	β	SE	Wald value	P value	OR	OR Lower limit (95% CI)	OR Upper limit (95% CI)
Age							
[<18]	0.849	0.823	1.065	0.302	2.338	0.466	11.738
Menche et al. (2015), Edinoff et al. (2021), Preskorn, 1997; Chi (2021), Wabont et al. (2022), Khalil et al. (2024), Jiang et al., 2024; Green (1988), Cosme et al. (1996), Friedenberg and Rothstein (1996), Cadranel et al. (1999), Odeh et al. (2001), Colakoglu et al. (2005), Pompili et al. (2008), Carvajal García-Pando et al. (2002), Hautekeete et al. (1998), Chen et al. (2020), Chen et al. (2023), Gleason et al. (2002), Schaefer et al. (2012), Teschke et al., 2025; Xu (2023), Huang et al. (2024), Baboota et al. (2022), Wei et al. (2024), Huang and Zhang (2008)	1.107	0.507	4.764	**0.029** ^**^	3.026	1.120	8.181
[≥65]	−0.093	0.562	0.028	0.868	0.911	0.303	2.741
Gender							
[F]	−0.113	0.402	0.079	0.779	0.893	0.406	1.965
Weight							
[≤60]	−0.793	0.470	2.852	**0.031** ^**^	0.453	0.180	1.136
[≥81]	−0.173	0.520	0.110	0.740	0.841	0.303	2.333
Dosage							
[Overdosing]	−0.377	0.462	0.665	0.415	0.686	0.277	1.697
[Underdosing]	−0.677	0.557	1.477	0.224	0.508	0.171	1.514

** P < 0.05, indicating a statistically significant association.

### 3.6 Construction of liver disease modules

**FIGURE 4:**
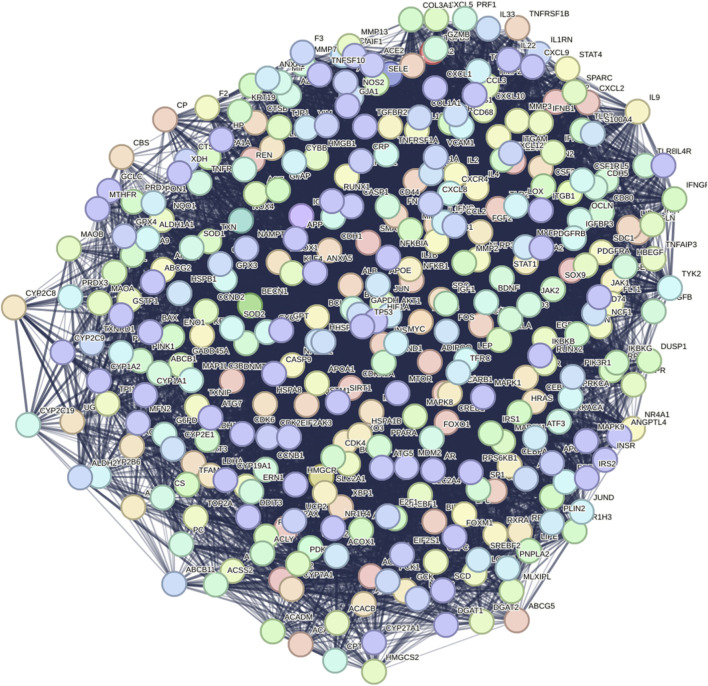
The liver disease module established for the signal of SSRIs-related serious adverse events of drug-induced liver injury (n = 852 nodes).

**FIGURE 5:**
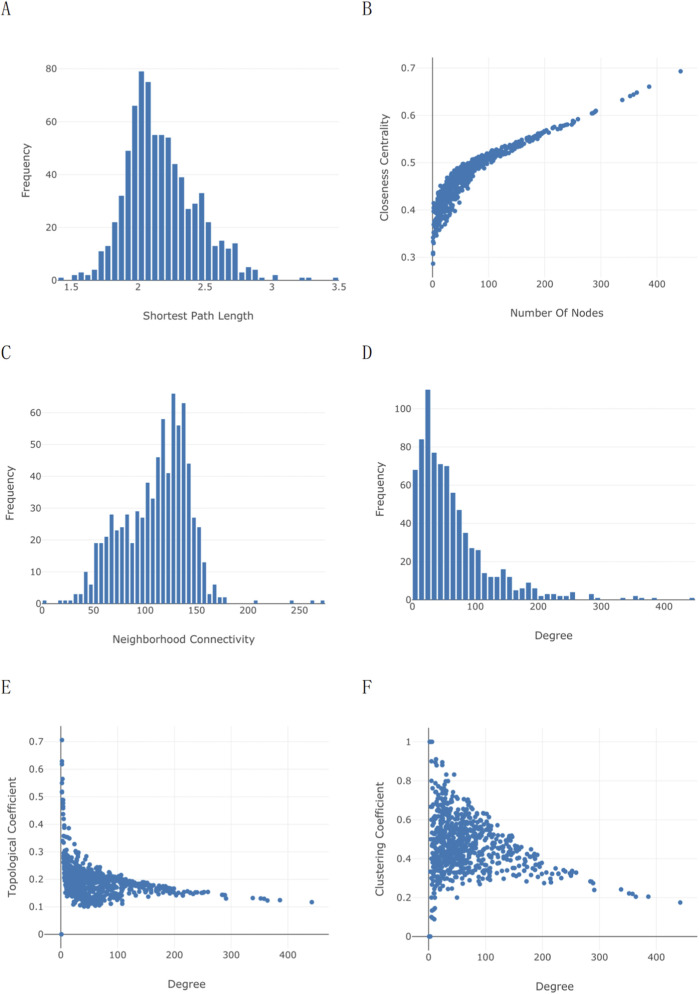
Topological characteristics of the liver disease module **(A)**, distribution of shortest path lengths (mean = 2.1, indicating high network connectivity); **(B)**, closeness centrality increases with node accumulation, suggesting centralized hub nodes; **(C)**, neighborhood connectivity predominantly between 50 and 150, indicating local clustering; **(D)**, degree distribution follows power-law (γ ≈ 2.3), characteristic of scale-free networks; **(E)**, inverse relationship between degree and topological coefficient; **(F)**, clustering coefficient declines with increasing degree, indicating hierarchical network organization.

Following the identification of four drugs associated with severe hepatic adverse drug reactions (ADRs), disease-related targets were collated from multiple pathological databases to establish a comprehensive liver disease module. After MeSH standardization, most curated disease terms corresponded to high-frequency ADRs. A total of 852 disease targets were ultimately retained. All collected disease targets and pharmacological targets were mapped onto the human interactome network derived from the STRING database, forming an integrated liver disease module (Menche et al., 2015) as depicted in Figure 4. To evaluate the biological regulatory pathway integrity and connectivity within the liver disease module, topological properties were analyzed using the Network Analyzer tool in Cytoscape 3.9 (Dai et al., 2020; Dai et al., 2020). As shown in Figure 5, the analytical framework incorporated six parameters: average shortest path length, closeness centrality, topological coefficient, shared neighbors, degree, and clustering coefficient. Results indicate that the module constitutes a sophisticated network system involving multiple targets and pathways. The constructed liver disease module demonstrates extensive coverage of hepatic phenotypes, consistent with the study’s objectives. This modular framework provides a robust foundation for mechanistic exploration of hepatotoxic disorders.

### 3.7 Topological characteristics of liver disease modules

As illustrated in Figure 4, Panel A displays the frequency distribution of shortest path lengths across all nodes, with the majority concentrated between 1.5 and 2.5, suggesting a highly interconnected network characterized by short average path lengths. Panel B depicts the relationship between node count and closeness centrality, where nodes with elevated closeness centrality exhibit enhanced accessibility to other network components. The progressive increase in closeness centrality with node accumulation indicates the presence of centralized hub nodes. Panel C presents the frequency distribution of neighborhood connectivity, quantifying the actual connections among a node’s immediate neighbors. Most nodes demonstrate neighborhood connectivity values between 50 and 150, indicative of localized clustering within the network. Panel D illustrates the degree frequency distribution, revealing a predominance of low-degree nodes with exponential decay in frequency as degree increases. This pattern suggests that a limited subset of highly connected proteins may serve as critical regulatory elements or functional hubs in biological processes. Panel E examines the correlation between node degree and topological coefficient, showing an inverse relationship where higher-degree nodes display reduced topological coefficients, implying diminished interconnectivity among their neighbors. Panel F analyzes the degree-clustering coefficient relationship, demonstrating a gradual decline in clustering coefficient with increasing node degree. The moderated rate of decrease suggests persistent local clustering even among high-degree nodes.

Collectively, these findings characterize the liver disease module as a sophisticated network architecture involving multiple molecular targets and pathways. Six topological metrics were systematically employed to assess the biological regulatory pathway integrity and connectivity within this module. The constructed framework demonstrates extensive coverage of hepatic phenotypic variations, aligning with the study’s objectives. This modular representation establishes a robust platform for mechanistic investigations into hepatotoxic disease pathogenesis, offering comprehensive insights into multiscale regulatory interactions.

### 3.8 Protein-protein interaction network analysis

**FIGURE 6:**
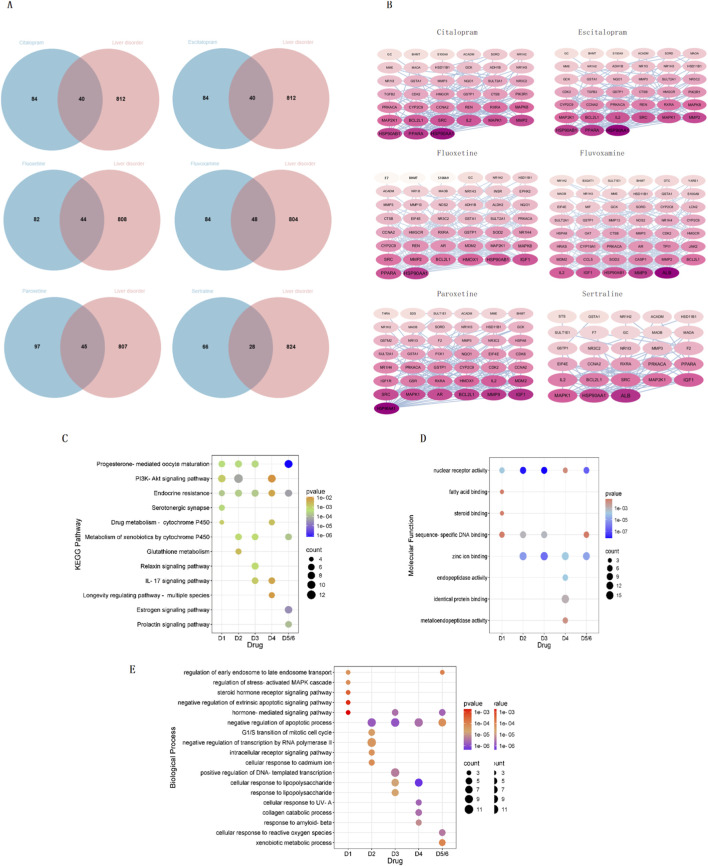
PPI networks and enrichment pathways of potential toxic targets for SSRI-DILI. **(A)** Venn diagram of the intersection targets of six SSRIs and the liver disease module. **(B)** PPI networks of intersection targets (nodes sized by degree). **(C)** KEGG top 5 enriched pathways of SSRIs drug-induced liver injury **(D)** MF top5 Enrichment Pathways for DILI Induced by Six SSRIs Drugs **(E)** BP top5 Enrichment Pathways for DILI Induced by Six SSRIs Drugs. (D1, sertraline, D2, paroxetine, D3, fluoxetine, D4, fluvoxamine, D5, escitalopram/citalopram).

The parameter *Ds* denotes the shortest path distance between drug targets and disease-associated targets, where *Ds* = 0 indicates overlapping targets between the disease module and pharmacological agents. As shown in Figure 6A, a Venn diagram illustrates the quantitative overlap between six SSRIs and the liver disease module. Escitalopram and citalopram share 40 overlapping targets with the module, while fluoxetine, fluvoxamine, paroxetine, and sertraline exhibit 44, 48, 45, and 28 overlapping targets, respectively.

Protein-protein interaction (PPI) networks were constructed for shared targets of each SSRI with the liver disease module using the STRING database and Cytoscape software (Figure 6B). Topological analysis identified HSP90AA1 as the highest-degree node in networks associated with citalopram, escitalopram, fluoxetine, and paroxetine, whereas ALB displayed maximal degree centrality in fluvoxamine and sertraline networks. These findings suggest potential toxicity-associated hubs underlying SSRI-induced drug-induced liver injury (DILI).

To investigate the biological roles of DILI-related toxicity targets across cellular processes, GO and KEGG pathway enrichment analyses were performed via the DAVID database. Following Bonferroni correction (FDR < 0.01), the top five enriched pathways are visualized in Figures 6C–E (D1: sertraline, D2: paroxetine, D3: fluoxetine, D4: fluvoxamine, D5: escitalopram/citalopram). Common mechanisms across five SSRIs predominantly involve endocrine regulation, cytochrome P450-mediated drug metabolism, and nuclear receptor activity modulation. Apoptosis inhibition pathways were implicated in all agents except sertraline. Distinct drug-specific pathway divergences emerged: sertraline engages PI3K-Akt signaling and stress-activated MAP kinase cascades; paroxetine associates with glutathione metabolism and PI3K-Akt signaling; fluoxetine links to IL-17-mediated inflammation and relaxin signaling; fluvoxamine enriches in IL-17 inflammatory pathways; escitalopram/citalopram activates estrogen signaling and reactive oxygen species responses.

This multi-modal analysis delineates both shared and unique molecular mechanisms underlying SSRI-associated hepatotoxicity, highlighting complex interplay between metabolic regulation, inflammatory cascades, and cellular survival pathways.

## 4 Discussion

### 4.1 Baseline characteristics analysis

Among reports of drug-induced liver injury (DILI) associated with six selective serotonin reuptake inhibitors (SSRIs), the highest number of cases occurred in patients aged 18–44 and 45–64, followed by elderly patients over 65 years, while the fewest reports involved individuals under 18. Although studies suggest an age-related increase in severe adverse drug reactions, large prospective DILI registries have not established age as an independent risk factor (Edinoff et al., 2021). However, cumulative age-related pharmacokinetic alterations and mitochondrial dysfunction in older adults have been proposed as potential contributors to higher DILI susceptibility. Additionally, reduced hepatic and renal clearance rates in the elderly, resulting in elevated plasma drug concentrations, may further amplify DILI risks (Preskorn, 1997).

The FAERS database revealed a significantly higher proportion of DILI cases in females than males. Nevertheless, the role of sex as a definitive susceptibility factor remains ambiguous. Research indicates that females may exhibit greater vulnerability to mild hepatic enzyme elevations during SSRI therapy, whereas males might experience higher risks of severe hepatotoxicity. The reported incidence of SSRI-related DILI correlates strongly with both population exposure levels and reporting patterns. While the precise influence of age, sex, and ethnicity on DILI susceptibility requires further clarification, clinicians should holistically assess individual patient profiles—particularly emphasizing risk evaluation in elderly populations and female patients—when prescribing SSRIs (Chi, 2021).

### 4.2 Adverse event signal analysis in SSRI-Induced DILI

Hepatocellular injury emerges as a shared adverse event signal across all six SSRIs, underscoring its role as a primary manifestation of SSRI-associated hepatotoxicity. Extensive studies demonstrate that hepatic metabolism of these drugs generates free radicals and toxic metabolites during biotransformation, directly contributing to hepatocellular damage (Wabont et al., 2022). This evidence reinforces the necessity for clinicians to closely monitor liver function parameters during SSRI therapy. Hepatocellular lysis represents a common adverse event signal for escitalopram, fluoxetine, paroxetine, and sertraline. Mechanistic investigations reveal that certain drug metabolites disrupt intracellular metabolic processes and compromise cell membrane integrity, ultimately inducing cytolysis (Khalil et al., 2024).

Individual SSRIs exhibit distinct adverse event profiles. Fluoxetine demonstrates unique hepatotoxic signals including hepatic steatosis, hyperbilirubinemia, and reduced coagulation factor VII levels. Post-marketing surveillance of fluoxetine documents reports of hepatic failure/necrosis, with prescribing information cautioning against its use in patients with cholestatic jaundice (Jiang et al., 2024; Green, 1988; NHSA, 2023). In cirrhotic populations, impaired clearance of fluoxetine and its active metabolite norfluoxetine results in prolonged elimination half-lives, necessitating dose reduction or extended dosing intervals (Cosme et al., 1996; Friedenberg and Rothstein, 1996). Paroxetine is exclusively associated with chronic active hepatitis, ammonia dysregulation, hepatomegaly, and hypertransaminasemia. Ammonia dysregulation reflects compromised detoxification capacity, and hepatomegaly/hypertransaminasemia indicates progressive hepatocellular damage (Cadranel et al., 1999). Clinical observational studies confirm these paroxetine-specific hepatic adverse events in long-term users (Odeh et al., 2001; Colakoglu et al., 2005; Pompili et al., 2008).

Sertraline displays a unique spectrum of severe hepatic adverse events, including primary biliary cholangitis, acute hepatitis, esophageal variceal hemorrhage, and hemorrhagic liver cysts. Among the six SSRIs, sertraline exhibits the highest frequency of hepatotoxicity signals, likely attributable to its widespread clinical use and large patient population, which increases the probability of adverse event detection and reporting (Carvajal García-Pando et al., 2002). The FDA’s quarterly watchlist highlights sertraline’s association with severe hepatic complications such as hepatitis, liver injury, and hepatic failure. Reported injury patterns encompass hepatocellular, cholestatic, or mixed types, with manifestations ranging from transient transaminase elevations to fulminant hepatitis (Hautekeete et al., 1998). Proactive monitoring of liver function is recommended, particularly during initial treatment phases or in high-risk populations (Chen et al., 2020). Citalopram is uniquely linked to congestive hepatopathy, suggesting its potential to alter hepatic hemodynamics. Citalopram may induce congestive hepatopathy by disrupting hepatic blood perfusion and venous return through abnormal vasoconstriction or dilation (Chen et al., 2023). Despite limited mechanistic studies, this distinct hemodynamic effect warrants clinical attention given its implications for hepatic circulatory integrity (Gleason et al., 2002; Schaefer et al., 2012). Among the DILI cases identified in our FAERS analysis, some may represent drug-induced autoimmune hepatitis (DIAIH), a distinct subtype that ideally requires dual causality assessment using RUCAM and the simplified AIH score (Teschke et al., 2025). Although FAERS lacks the serological and histological data needed to apply these criteria, signals for chronic hepatitis (e.g., with paroxetine) and previous case reports linking fluoxetine and paroxetine to DIAIH suggest this possibility warrants further investigation.

### 4.3 Analysis of onset timing in SSRI-induced DILI

Literature review indicates a temporal pattern in SSRI-induced drug-induced liver injury (DILI) onset. Most DILI events occur between several days and 6 months after initiating antidepressant therapy, correlating with drug metabolic characteristics and hepatic response timelines (Preskorn, 1997). For instance, sertraline- and escitalopram-associated DILI typically manifests within 15 days to 6 months of treatment, while fluoxetine exhibits a latency period of 5–90 days. Paroxetine-related hepatic abnormalities are frequently detected during early treatment phases (2–8 weeks) (Xu, 2023). These findings suggest that SSRI hepatotoxicity may arise from cumulative drug effects, particularly through prolonged exposure to toxic metabolites during hepatic metabolism (Khalil et al., 2024). Analysis of FAERS data in this study aligns with these patterns, revealing concentrated adverse event (AE) onset within the first treatment month.

Age-specific variations in DILI onset were statistically significant (P < 0.01). The 18–44 age group demonstrated the shortest median onset time (22 days), contrasting with the longest interval in the under-18 cohort (45 days) (Huang et al., 2024). This divergence likely reflects age-dependent hepatic metabolic capacity, as aging livers undergo structural and functional decline, including reduced volume, lipofuscin accumulation, diminished Phase I metabolism, altered protein expression, and impaired hepatobiliary function. Additionally, aging exacerbates susceptibility to insulin resistance, oxidative stress, apoptosis, and cytokine dysregulation, further compromising hepatic regeneration and promoting disease progression (Baboota et al., 2022). Enhanced metabolic efficiency in younger adults may accelerate hepatotoxic manifestation, whereas underdeveloped enzymatic systems in pediatric populations prolong drug accumulation, delaying clinical detection (Wei et al., 2024). Despite reduced metabolic capacity in elderly patients, the 65+ age group exhibited relatively rapid median onset (29 days), potentially indicating heightened drug toxicity sensitivity in aged livers (Huang and Zhang, 2008).

Outcome-stratified analysis revealed significant onset time disparities (P < 0.01). Fatal cases showed the longest median onset (59.5 days), while life-threatening events had the shortest (12.5 days). Although no prior studies explain this pattern, it may reflect injury progression dynamics: mild hepatic injuries may be detected and managed promptly, whereas severe injuries require prolonged progression to reach life-threatening stages. Hospitalized patients displayed a median onset of 20 days, suggesting effective early clinical recognition and intervention, likely attributable to timely medical monitoring (Xu et al., 2023).

### 4.4 Univariate logistic regression analysis of mortality outcomes in patients with SSRI-induced DILI

Analysis of the FAERS database revealed significantly higher mortality risk in patients aged 18–44 compared to those aged 45–65 (OR = 3.026, P < 0.05), potentially attributable to accelerated DILI progression from elevated basal metabolic rates and rapid drug clearance in younger individuals. Elderly patients (>65 years) exhibited increased DILI susceptibility due to comorbidities such as chronic liver disease and cardiovascular conditions, which independently elevate hepatotoxicity risks (Cui et al., 2025). These findings underscore the necessity for enhanced hepatic monitoring in both young and geriatric populations during SSRI therapy.

Univariate logistic regression identified body weight as a critical determinant of DILI outcomes, with patients weighing <60 kg demonstrating significantly lower mortality risk than the 61–80 kg group (OR = 0.453, P < 0.05). While extreme BMI values and obesity-related metabolic syndrome correlate with elevated DILI mortality (Cui et al., 2025), current evidence lacks conclusive data linking body weight directly to fatal outcomes, necessitating large-scale real-world studies to clarify this relationship (Cao et al., 2025). Weight-adjusted dosing strategies remain clinically recommended to mitigate hepatotoxicity risks.

Sex-specific analyses yielded divergent insights. Although observational studies suggest females may experience more frequent mild transaminase elevations and males higher severe liver injury rates (Todorović Vukotić et al., 2021), FAERS data showed no statistically significant sex-based differences in DILI mortality. This indicates sex may not serve as an independent prognostic factor in SSRI-induced hepatotoxicity. Nevertheless, physiological variations in drug metabolism and hormonal influences warrant continued consideration during therapeutic decision-making (Preskorn, 1997).

Age and body weight emerged as pivotal modifiable risk factors for DILI outcomes, while sex and dosage showed limited predictive value. Clinical practice should prioritize systematic evaluation of age, weight, and comorbidities when prescribing SSRIs, coupled with individualized dosing and monitoring protocols. Although SSRI-associated hepatotoxicity remains uncommon, prompt detection of transaminase elevations mandates rigorous hepatic surveillance, particularly in vulnerable age groups.

### 4.5 Hypothesized shared toxicological mechanisms underlying SSRI-associated DILI

This study identified convergent hepatotoxic mechanisms across SSRIs despite their structural heterogeneity through network toxicological analysis. Enrichment analysis revealed three shared molecular pathways: cytochrome P450-mediated xenobiotic metabolism, negative regulation of apoptosis, and nuclear receptor activity.

CYP450 metabolic disruption initiates oxidative stress cascades. All SSRIs undergo hepatic metabolism via cytochrome P450 isoforms (CYP2D6, CYP3A4), generating electrophilic intermediates that impair mitochondrial respiratory chain complexes and reduce ATP synthesis efficiency (Zhao et al., 2017; Czarny et al., 2018). This predicted mechanism would align with FAERS-reported signals of hepatic steatosis and hyperbilirubinemia, as suppressed β-oxidation might promote lipid accumulation and uncoupled oxidative phosphorylation could exacerbate bilirubin dysregulation (Shen and Hammock, 2012).

Apoptotic network dysregulation represents a common hepatotoxic endpoint. We hypothesize that SSRIs may disrupt the balance of Bax/Bcl-xL expression ratios, potentially altering mitochondrial membrane potential (Guo et al., 2002; Nabekura et al., 2020). Concurrent endoplasmic reticulum (ER) stress might amplify apoptosis through unfolded protein response (UPR) activation, where misfolded protein accumulation upregulates pro-apoptotic factors (Bi et al., 2022; Levada et al., 2018). HSP90AA1 depletion correlates with Bax elevation despite lacking direct interaction, while elevated ER stress markers implicate chaperone dysfunction in UPR initiation (Lebeaupin et al., 2018; Mun et al., 2013). This hypothetical mechanism corresponds to FAERS-observed hypocoagulability, as ER stress could theoretically inhibit hepatic coagulation factor synthesis (Bilici et al., 2001).

Nuclear receptor modulation exerts dual hepatotoxic effects. PXR activation induces compensatory CYP3A4 overexpression, transiently enhancing detoxification but ultimately promoting toxic intermediate accumulation through metabolic saturation (Wang et al., 2023). This “compensation-decompensation” transition may explain elevated adverse event rates for sertraline and citalopram in FAERS. Conversely, FXR suppression downregulates bile salt export pump (BSEP) expression, causing hydrophobic bile acid retention and cholestatic injury (Wang et al., 2020). These mechanisms potentially underlie sertraline-associated primary biliary cholangitis and citalopram-linked congestive hepatopathy through biliary epithelial damage and hepatic hemodynamic disturbances (Hu et al., 2024).

These multilayered mechanisms—metabolic activation, apoptotic cascade amplification, and nuclear receptor-mediated regulation—collectively establish the molecular foundation for SSRI hepatotoxicity, reconciling clinical observations with mechanistic insights from network toxicology analysis.

### 4.6 Exposure bias and its impact on signal interpretation

A critical limitation of spontaneous reporting systems like FAERS is the absence of denominator data—specifically, the total number of prescriptions dispensed for each medication. This precludes calculation of true incidence rates and introduces significant exposure bias when comparing raw report counts across drugs. Sertraline had the highest number of DILI reports (n = 463) among the six SSRIs, followed by citalopram (n = 429) and fluoxetine (n = 306), while fluvoxamine had the fewest (n = 9). However, these figures must be interpreted in the context of prescribing patterns.

The low number of fluvoxamine reports (n = 9) likely reflects both lower prescription volume and possible underreporting, as fluvoxamine is less commonly used for depression and more frequently prescribed for obsessive-compulsive disorder. Conversely, sertraline’s high report count may partly reflect its widespread use rather than intrinsically greater hepatotoxicity. Despite this limitation, the distinct adverse event profiles identified for each drug (e.g., fluoxetine with steatosis, paroxetine with chronic hepatitis) are less susceptible to exposure bias. Therefore, future pharmacovigilance studies should integrate prescription databases (e.g., IQVIA, CPRD) to calculate reporting rates of prescriptions, enabling more accurate comparisons of hepatotoxicity risk across SSRIs.

## 5 Limitations

While our study offers significant insights into the hepatotoxicity linked to SSRIs, it is crucial to recognize its limitations. The FAERS database exhibits inherent limitations primarily stemming from its voluntary reporting system, which may introduce reporting biases and incomplete data capture. And without access to complete medical records, we could not apply a structured causality assessment method like the RUCAM to each individual report, which is considered the standard for definitive DILI diagnosis. As a result, our study cannot determine what proportion of the reported ‘DILI’ cases were actually due to other conditions. The absence of prescription denominator data in FAERS precludes calculation of true incidence rates. The higher number of reports for sertraline compared to fluvoxamine may reflect prescribing volume differences rather than differential hepatotoxicity. While disproportionality analysis partially mitigates this bias, it does not eliminate it entirely. A further limitation is our inability to distinguish between classic DILI and DIAIH in the FAERS data, a distinction that is clinically important and methodologically challenging. Our definition of DILI relied on the MedDRA SMQ for severe liver events, which is based on reported preferred terms rather than standardized biochemical criteria (e.g., ALT >3× upper limit of normal, ALP >2× ULN, or R value calculation). The absence of laboratory data in FAERS precludes application of the internationally accepted DILI consensus criteria. Consequently, some cases included may not meet strict biochemical thresholds for DILI, and misclassification is possible. We have therefore used the broad SMQ definition to maximize sensitivity at the cost of specificity, and our findings should be interpreted as signals requiring confirmation with laboratory-adjudicated DILI cases. Notably, mild or non-severe cases are likely underreported, creating inherent data imbalances (Blau et al., 2017). Geographical representation disparities further constrain generalizability, with predominant data from Western populations and insufficient contributions from Asian regions. Methodological constraints include the retrospective nature of FAERS analyses, where observational designs facilitate pattern identification but preclude causal inference (Fadini et al., 2018). While network pharmacology approaches effectively map potential SSRI-gene interactions in drug-induced liver injury, these computational predictions require empirical validation. Future investigations should prioritize experimental confirmation of predicted molecular targets through integrated approaches combining *in vitro* models, animal studies, and multi-ethnic clinical cohorts to establish mechanistic causality. Such translational validation will strengthen the biological plausibility of network-derived hypotheses and enhance the clinical relevance of pharmacogenomic findings.

## 6 Conclusion

This study comprehensively analyzed DILI risks associated with six SSRIs using big-data methodologies. The high incidence of SSRI-related DILI during the first month of treatment highlights the need for enhanced liver function monitoring during initial therapy, particularly in female patients. Significant variability in hepatotoxicity profiles among SSRIs necessitates differentiated monitoring protocols based on specific drugs. The association between onset time and prognosis suggests vigilance for early liver injury signals in younger patients, while delayed onset may indicate poor outcomes, warranting dynamic monitoring systems and extended observation periods for elderly patients. Elucidation of DILI mechanisms—including CYP450 dysfunction, oxidative stress, and drug-specific pathways—supports individualized therapeutic decisions. By integrating big-data analytics with multi-omics insights, this study establishes a scientific foundation for optimizing treatment regimens and developing risk management guidelines, offering critical theoretical support for clinical personalized medicine and drug safety evaluation.

## Data Availability

The original contributions presented in the study are included in the article/supplementary material, further inquiries can be directed to the corresponding authors.
